# Type 2 diabetes and obesity induce similar transcriptional reprogramming in human myocytes

**DOI:** 10.1186/s13073-017-0432-2

**Published:** 2017-05-25

**Authors:** Leif Väremo, Tora Ida Henriksen, Camilla Scheele, Christa Broholm, Maria Pedersen, Mathias Uhlén, Bente Klarlund Pedersen, Jens Nielsen

**Affiliations:** 1 Department of Biology and Biological Engineering, Chalmers University of Technology, 41296, Gothenburg, Sweden; 2 Centre of Inflammation and Metabolism and Centre for Physical Activity Research, Rigshospitalet, University of Copenhagen, 2100, Copenhagen Ø, Denmark; 3 Novo Nordisk Foundation Center for Basic Metabolic Research, University of Copenhagen, 2200, Copenhagen N, Denmark; 4 Department of Proteomics, School of Biotechnology, AlbaNova University Center, Royal Institute of Technology (KTH), 10691, Stockholm, Sweden; 5 Science for Life Laboratory, Royal Institute of Technology (KTH), 17121 Stockholm, Sweden

**Keywords:** Type 2 diabetes, Obesity, Skeletal myocytes, RNA-seq, Gene expression, Gene-set analysis, Metabolic network

## Abstract

**Background:**

Skeletal muscle is one of the primary tissues involved in the development of type 2 diabetes (T2D). The close association between obesity and T2D makes it difficult to isolate specific effects attributed to the disease alone. Therefore, here we set out to identify and characterize intrinsic properties of myocytes, associated independently with T2D or obesity.

**Methods:**

We generated and analyzed RNA-seq data from primary differentiated myotubes from 24 human subjects, using a factorial design (healthy/T2D and non-obese/obese), to determine the influence of each specific factor on genome-wide transcription. This setup enabled us to identify intrinsic properties, originating from muscle precursor cells and retained in the corresponding myocytes. Bioinformatic and statistical methods, including differential expression analysis, gene-set analysis, and metabolic network analysis, were used to characterize the different myocytes.

**Results:**

We found that the transcriptional program associated with obesity alone was strikingly similar to that induced specifically by T2D. We identified a candidate epigenetic mechanism, H3K27me3 histone methylation, mediating these transcriptional signatures. T2D and obesity were independently associated with dysregulated myogenesis, down-regulated muscle function, and up-regulation of inflammation and extracellular matrix components. Metabolic network analysis identified that in T2D but not obesity a specific metabolite subnetwork involved in sphingolipid metabolism was transcriptionally regulated.

**Conclusions:**

Our findings identify inherent characteristics in myocytes, as a memory of the in vivo phenotype, without the influence from a diabetic or obese extracellular environment, highlighting their importance in the development of T2D.

**Electronic supplementary material:**

The online version of this article (doi:10.1186/s13073-017-0432-2) contains supplementary material, which is available to authorized users.

## Background

Type 2 diabetes (T2D) is a complex metabolic disease characterized by increased resistance to insulin in several tissues, including liver, adipose tissue, and skeletal muscle, leading to abnormally high blood glucose levels and compromised pancreatic insulin secretion [1]. The incidence of the disease is increasing with epidemic rates and is currently affecting around 350 million people [2]. Contributing to the complexity of T2D, a range of risk factors are associated with and influence the development of this disease, including genetic and epigenetic components, sedentary lifestyle, diet, and obesity [3]. Indeed, most T2D subjects are obese [4] and the co-occurrence of these two conditions makes it difficult to study their individual effects on the cell, in particular that of T2D. There is also evidence of differences in the onset of T2D in obese and non-obese subjects [5]. Collectively, the co-occurrence of obesity in T2D subjects hinders the ability to pinpoint what truly underlies the etiology of the disease.

Skeletal muscle is responsible for around 75% of the glucose uptake that is stimulated by insulin and thus has a big impact on glucose homeostasis [6, 7]. Insulin resistance in skeletal muscle is considered to be one of the early key defects in the development of T2D [8]. It is therefore critical to improve our understanding of the development of insulin resistance and the molecular mechanisms taking place in skeletal muscle as a contributing cause or consequence of T2D. To accomplish this in a controlled manner, primary differentiated myotubes, a well-described in vitro model of human skeletal muscle, can be used [9]. These cells maintain many features of the donor phenotype, including genetic and epigenetic components, and, importantly, several properties related to the T2D phenotype have been shown to be conserved in the in vitro myocyte model [9–14].

Here we implemented a unique experimental design, using the in vitro myocyte system, which enabled us to identify and characterize intrinsic properties of myocytes in association with T2D and obesity, independently. The detected changes represent properties that are inherited from muscle precursor cells of the subjects and retained in myocytes cultured in an environment without influence from external factors related to the in vivo phenotype (e.g., varying levels of hormones and cytokines). Therefore, these properties are hard-wired in their association to T2D and obesity, and are not a response to the influence of the surrounding phenotype. In this study, we sought to analyze these properties in an attempt to identify molecular drivers of T2D in the skeletal muscle.

## Methods

### Subjects and phenotype measurements

Participants (*n* = 24) were selected from a larger cohort [15] and divided into four groups based on their body mass index (BMI) and the results of an oral glucose tolerance test: normal glucose tolerant (NGT)/non-obese; NGT/obese; T2D/non-obese; and T2D/obese. The four groups are from here on referred to as controls, OB, T2D, and T2D&OB, respectively. Subjects gave their informed consent and the study was approved by the Ethical Committee of Copenhagen and Frederiksberg Council, Denmark. Before the experimental day, all participants underwent a clinical examination with blood samples. See Additional file 1: Supplementary material and methods for more details.

### Culturing of myocytes and sampling of RNA for RNA-sequencing

Human muscle biopsies were taken from the vastus lateralis muscle with a modified Bergström needle (including suction) [16] under local anesthesia with 2% lidocaine, as described in detail previously [11]. Muscle precursor cells (satellite cells) were isolated and cultured in growth media and plated in six-well plates for differentiation (see Additional file 1: Supplementary methods for details). Cultures were fully differentiated at day 5 (>3 nuclei per myotube in ~70% of the cells). The myotubes were stimulated with insulin (100 nM) and harvested after 0 (before insulin stimulation), 0.5, 1, or 2 h. Total RNA was extracted using TRIzol® (Life Sciences) according to the manufacturer’s instructions and purified by poly(A) enrichment using Illumina TruSeq RNA.

### RNA-sequencing and data preprocessing

The RNA samples were sequenced with Illumina HiSeq 2000 and 2500 instruments, generating on average 68 million paired-end reads per sample. RNA-seq data for the six base-line (0 h) control samples were already published and are available at the Gene Expression Omnibus (GSE63887). The remaining data for 90 samples have been deposited under the accession number GSE81965. The reads were trimmed from adapter sequences and aligned to the human genome (Ensembl GRCh37.73, DNA primary assembly) using STAR 2.3.1u [17] and the corresponding Ensembl gene structure gtf file. The resulting bam files were indexed and sorted using Samtools 0.1.18 [18]. As input for the differential expression analysis, gene counts were calculated using HTSeq-count 0.5.4p3 [19] with default settings.

### Differential expression analysis

The Bioconductor [20] R-package limma [21] was used for differential expression analysis. First, genes were filtered according to a cutoff of CPM >0.3 in at least six samples (roughly equivalent to a read count of 20). Weighted trimmed mean of M-values (TMM) normalization [22] was performed using edgeR [23] and the data were then passed to the voom function in limma [24]. The duplicateCorrelation function was used to handle the correlation of samples originating from the same subjects [25]. Finally, the limma workflow for differential expression analysis was run, using linear modeling and empirical Bayes statistics, implemented in the functions lmFit and eBayes [26] (see Additional file 1: Supplementary methods for details). All differential expression analysis results are available in Additional file 2: Table S1. Correction for multiple testing was performed by adjusting the *p* values to control the false discovery rate (FDR) according to the method described by Benjamini and Hochberg [27] as implemented in the p.adjust function in R. The adjusted *p* values are referred to as *q* values.

### Gene-set analysis

All gene-set analyses (GSAs) were carried out using the Bioconductor R package piano [28]. Gene-set *p* values were calculated using gene-wise permutation (10,000 times) and *p* values were adjusted for multiple testing (*q* values) in the same manner as for the gene-level *p* values. Consensus GSA was used by running two GSAs in parallel, first with gene-level *q* values and second with log2 fold changes as gene-level statistics. The results were combined so that average *q* values were obtained for each gene-set, thus favoring gene-sets with genes displaying both large fold changes and high statistical significance. Significant gene-sets were selected using a cutoff of (average) *q* < 0.001 (for the so called non-directional class). See Additional file 1: Supplementary methods for details.

### Reporter metabolite analysis

Metabolite gene-sets were acquired from the myocyte genome-scale metabolic network iMyocyte2419 [29]. Reporter metabolite analysis was performed using the reporter features algorithm [30] implementation in piano. To avoid unspecific metabolite gene-sets, only gene-sets with 3–50 genes were used in the analysis. Gene-set *p* values were adjusted for multiple testing (*q* values) in the same way as for the other GSAs. The python package Kiwi [31] was used through the BioMet Toolbox interface [32] for visualizing the significant metabolites and their interactions, using a significance cutoff of *q* < 0.003 and a shortest path length cutoff of 2. High-degree metabolites (>73 edges) were removed from the myocyte metabolic network before visualization. This did not remove any significant metabolites, but disconnected oleoyl-CoA from the subnetwork which otherwise would have been connected through a high-degree metabolite or co-factor.

### eQTL analysis

The NCBI GTex eQTL Browser [33] was used for acquiring eQTLs associated with obesity and T2D. These data were integrated with our results to identify genes that were both an eQTL and significantly differentially expressed. See Additional file 1: Supplementary methods for details.

### Additional subjects, RNA isolation, and quantitative PCR

In vitro myocytes, cultured as described above (but excluding insulin stimulation), from an additional set of 12 male subjects (from the same larger cohort as the 24 main subjects, described above) were used for follow-up analysis using quantitative real-time PCR (qPCR). Four of the original 24 subjects were also included (one T2D/non-obese and three NGT/non-obese males), in total summing up to 16 samples (eight T2D + eight healthy controls) taken at baseline, i.e., before/without insulin stimulation (see Additional file 3: Table S2 for subject characteristics). Total RNA was extracted from myocytes using TRIzol according to the manufacturer’s instructions. Total RNA was dissolved in RNase-free water and quantified using a Nanodrop ND 1000 (Saveen biotech ApS, Arhus, Denmark). Total RNA (500 ng) was reverse transcribed using the High Capacity Reverse Transcription kit (Applied Biosystems, Foster City, CA, USA) according to the manufacturer’s protocol. qPCR was performed in triplicate using the ViiA™ 7 Real-Time PCR platform. The primer sequences are listed in Additional file 4: Table S3. Data analysis was performed using the comparative method (ΔΔCT). *PPIA* was utilized as an endogenous control.

## Results

### Characterizing inherent properties of myocytes using RNA-seq

**Fig. 1:**
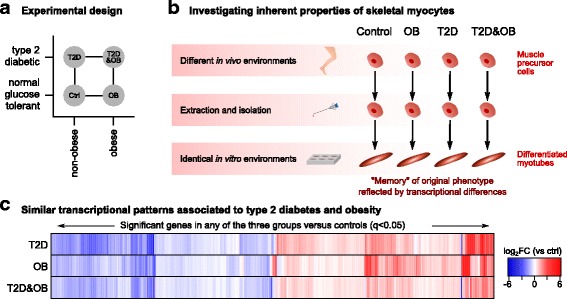
High similarity between the inherent transcriptional profiles associated with obesity and T2D. **a** The factorial design used to study T2D and obesity. Each group consists of three females and three males. **b** Muscle precursor cells were isolated from the subjects and differentiated in vitro. The cells were stimulated with insulin and samples for RNA-seq were taken at 0, 0.5, 1, and 2 h after stimulation in order to detect inherent transcriptional profiles associated with the four groups. **c** Heatmap of the fold changes for genes that were significant in at least one of the three groups compared to controls. Values are hierarchically clustered (dendrogram not shown), displaying the high consistency between the transcriptional signatures of the T2D, OB, and T2D&OB groups. (See also Additional file 5: Figure S1 and Additional file 6: Figure S2)

To study the individual effects of T2D and obesity we used a factorial design with two levels of each of the main factors (T2D and obesity). This resulted in four subject groups, as shown in Fig. 1a: controls (NGT and non-obese subjects), the T2D group (T2D but non-obese subjects), the OB group (obese but NGT subjects), and finally the T2D&OB group (subjects being both obese and T2D). Obesity was defined as having a BMI of more than 30 kg/m^2^. According to these criteria a total of 24 human subjects (three males and three females per group) were selected from a larger cohort, previously described by Pedersen et al. [15]. The subject characteristics are presented in Table 1. Muscle precursor cells from the subjects were isolated and differentiated in vitro, under identical conditions, into myotubes (subsequently referred to as in vitro myocytes). In principle, the only distinction between the different in vitro myocytes is that they are derived from precursor cells from subjects with different phenotypes and genotypes (Fig. 1b). Thus, if differences can be detected between in vitro myocytes from the different groups, those will represent properties that are intrinsic to the cells and not directly related to the influence from varying in vivo extracellular factors.

**Table 1:** Subject characteristics for the RNA-seq dataset

	Control	OB	T2D	T2D&OB	*P* value
Sex	3 M/3 F	3 M/3 F	3 M/3 F	3 M/3 F	-
Age, years	48–63	41–56	50–63	46–63	0.155
BMI, kg/m^2^	24.0 ± 0.6^b,d^	35.2 ± 3.6^a,c^	24.4 ± 2.7^b,d^	33.2 ± 2.8^a,c^	2.04e-07
Fasting glucose (0 h), mmol/l	5.4 ± 0.6^c^	5.7 ± 0.5	9.0 ± 3.8^a^	8.1 ± 1.4	0.0156
Glucose (2 h), mmol/l	5.2 ± 1.0^c,d^	5.5 ± 1.2^c,d^	18.0 ± 7.3^a,b^	16.4 ± 3.5^a,b^	1.12e-05
Insulin (0 h), pmol/l	25 ± 7^d^	60 ± 33	38 ± 35	79 ± 43^a^	0.0438
Insulin (2 h), pmol/l	175 ± 123	197 ± 153	281 ± 303	509 ± 376	0.137
HOMA-IR	0.47 ± 0.13^d^	1.13 ± 0.61	0.81 ± 0.72	1.61 ± 0.83^a^	0.0347
Cholesterol (total), mmol/l	5.2 ± 0.8	4.9 ± 0.3	5.1 ± 0.7	5.0 ± 0.6	0.918
Cholesterol (HDL), mmol/l	1.7 ± 0.5	1.3 ± 0.3	1.4 ± 0.4	1.2 ± 0.3	0.202
Cholesterol (LDL), mmol/l	2.8 ± 0.5^d^	3.4 ± 0.6	3.1 ± 0.4	3.6 ± 0.5^a^	0.0512
Triglycerides, mmol/l	1.0 ± 0.2	1.2 ± 0.8	1.7 ± 0.8	2.1 ± 0.7	0.0586
Free fatty acids (0 h), mmol/l	0.31 ± 0.09^c,d^	0.44 ± 0.13	0.56 ± 0.22^a^	0.61 ± 0.07^a^	0.0078
Free fatty acids (2 h), mmol/l	0.07 ± 0.03	0.07 ± 0.04	0.13 ± 0.05	0.13 ± 0.03	0.0182

Ages are shown as ranges, other values are means ± standard deviations. Differences between means were compared using one-way ANOVA, and post-hoc Tukey’s test was used for testing pair-wise group differences. Superscripts denote significant difference (*p* < 0.05) from a) Control, b) OB, c) T2D, and d) T2D&OB. *P* values in last column are from the ANOVA F-test

A hallmark of T2D is increased insulin resistance. In order to be able to capture both baseline transcriptional differences and potential differences in transcriptional responses to insulin, the in vitro myocytes were stimulated with insulin and samples for RNA-seq were taken at baseline and at 0.5, 1, and 2 h after stimulation, resulting in a total of 96 samples.

We performed differential expression analysis using a linear model that could capture the main effects of T2D and obesity, as well as their possible influence on each other. This approach enabled the comparison of the four subject groups and also included factors adjusting for the potential influence on gene expression by insulin, sex, and age. The complete differential expression results, including FDR-adjusted *p* values (*q* values) and fold changes, are available in Additional file 2: Table S1.

### Inherent transcriptional profiles associated with T2D and obesity are remarkably similar

Insulin had a significant effect on transcription, but this effect did not differ between the four groups (Additional file 5: Figure S1). Next, the transcriptional signatures associated with T2D and obesity were assessed by comparing the T2D, OB, and T2D&OB groups to controls. Distinctive transcriptional changes were detected in these comparisons (Additional file 6: Figure S2a), confirming the existence of inherent properties of myocytes derived from subjects with T2D and/or obesity. These findings were robust with regards to using different combinations of factors in the linear models (Additional file 5: Figure S1).

Hierarchical clustering of the fold changes of the significant genes in at least one of the three comparisons revealed a notable similarity in the transcriptional patterns of the T2D, OB, and T2D&OB groups (Fig. 1c). The Pearson correlation of the fold changes was also high (0.67, 0.81, and 0.65) between the three groups (Additional file 6: Figure S2b). It is remarkable that the inherent transcriptional profile associated specifically with T2D (without the influence of obesity) was consistent with the profile associated specifically with obesity (without the influence of T2D). This pattern is apparently also conserved in in vitro myocytes derived from subjects that are both obese and diabetic.

In addition, we correlated expression patterns and sequence variations in our data, based on known expressed quantitative trait loci, and identified seven genes (*PPARG*, *FBN2*, *JAZF1*, *ANXA5*, *RFTN1*, *IRS1*, and *MACROD2*) potentially influenced by short nucleotide polymorphisms (Additional file 7: Figure S3).

### The transcriptome data indicate an influence from the specific histone methylation H3K27me3

**Fig. 2:**
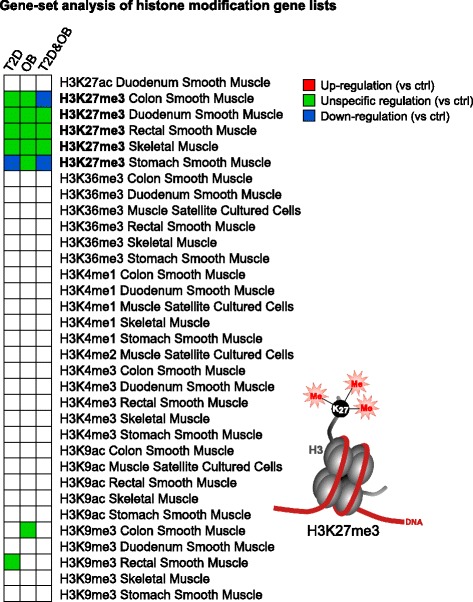
All investigated histone modification gene-sets. Significant gene-sets are marked as either *red* (up-regulated), *blue* (down-regulated), or *green* (unspecifically regulated). The sets consisting of genes with H3K27me3 marks (determined by separate ChIP-seq experiments) were enriched by significantly differentially expressed genes, implying that there may be an influence on the observed transcriptional patterns, mediated through this specific histone methylation. (See also Additional file 8: Figure S4)

The presence of inherent transcriptional patterns is likely mediated by some combination of genetic and epigenetic mechanisms. To investigate whether there could be any influence from histone modifications we performed gene-set analysis (GSA), using gene-sets acquired from the Epigenomics Roadmap project, to identify histone modification gene-sets that were enriched by differentially expressed genes. One histone modification turned out to be significant for all three groups (T2D, OB, and T2D&OB), namely tri-methylation of lysine 27 on histone 3 (H3K27me3), which was represented by five gene-sets (Fig. 2). To validate these results, we repeated the analysis using histone modification gene-sets from the ENCODE project, confirming the significance of the H3K27me3 modification (Additional file 8: Figure S4a). There was a slight indication of down-regulation for some of the gene-sets, but in general the H3K27me3 gene-sets showed unspecific regulation, i.e., genes that have been shown to be associated with H3K27me3 displayed a mix of up- and down-regulation in our data. In addition, we calculated the number of significant genes among the H3K27me3 genes and for randomly permuted genes sets of the same size (Additional file 8: Figure S4b). Clearly there is a substantially higher portion of H3K27me3 genes that are transcriptionally regulated in T2D, OB, and T2D&OB (compared to controls) than expected by random chance.

H3K27 methylation patterns can be inherited from the muscle precursor cells, but also be affected by the activity of histone methyltransferases and demethylases in the in vitro myocytes. The H3K27me3 demethylases *KDM6A* (*UTX*) and *KDM6B* (*JMJD3*) [34] did not show significant changes in our differential expression results. The PRC2 complex is involved in H3K27 methylation involving the subunits EZH2, RBBP4 (RbAp48), RBBP7 (RbAp46), SUZ12, and EED [35]. Of these, only *RBBP4* was found significantly differentially expressed (*q* = 0.002) displaying a down-regulation in T2D.

### Functional characterization of the inherent transcriptional signatures

**Fig. 3:**
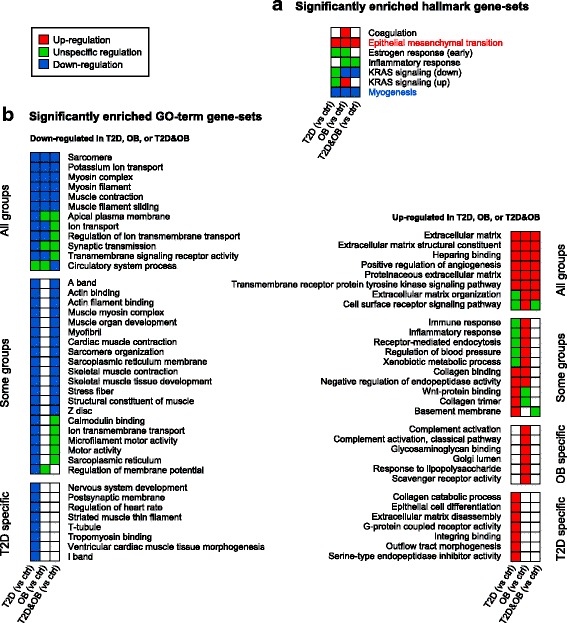
Functional characterization of the transcriptional signatures. **a** Significant hallmark gene-sets (see also Additional file 9: Figure S5). **b** Significantly up-regulated and down-regulated Gene Ontology (*GO*) terms (see Additional file 10: Figure S6 for GO terms with unspecific regulation)

To characterize the functions represented by the observed gene expression profiles we started by exploring a collection of 50 so-called hallmark gene-sets that have been computationally and manually curated, refined, and validated [36]. The GSA results for the T2D, OB, and T2D&OB groups (versus controls) were quite consistent for all three groups and clearly indicated two significant processes that were top ranked in all groups and showed a consistent distinct regulation. First, up-regulation of epithelial–mesenchymal transition (EMT), and second, down-regulation of myogenesis (Fig. 3a; Additional file 9: Figure S5).

To complement this analysis we also ran a Gene Ontology (GO) term [37] GSA. These results also supported the observed consistency in transcriptional profiles of T2D and obesity, in terms of a high extent of overlapping significant gene-sets between the three groups, but also highlighted some group-specific GO terms (Fig. 3b; Additional file 10: Figure S6). The results indicated up-regulation of genes involved in inflammatory and immune response (in line with the hallmark gene-sets), and in processes involved in the structure and function of the extracellular matrix. The down-regulated GO terms were almost exclusively related to muscle cell function and structure, in line with the observed down-regulation of the myogenesis hallmark gene-set. On the gene level, there was also a significant (*q* < 0.05) down-regulation of the myogenic markers *MYOD1*, *MYOG*, *TNNI1*, *MYH2*, and *MEF2C* in the T2D group (see details in Additional file 2: Table S1). *MEF2C* was also significantly down-regulated in the OB and T2D&OB groups.

### Network-dependent analysis reveals changes in sphingolipid metabolism in association with T2D

**Fig. 4:**
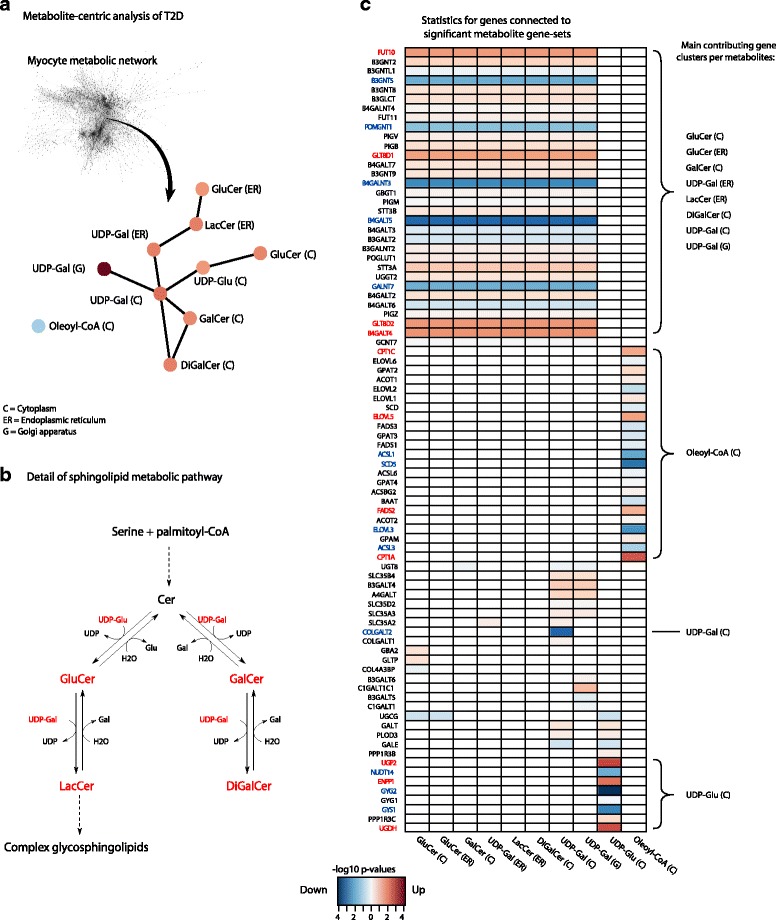
Reporter metabolite analysis of T2D. **a** Reporter metabolite analysis was run to identify metabolites significantly affected by differential expression. This analysis revealed a tightly connected subnetwork of metabolites participating in sphingolipid metabolism that is affected by transcriptional regulation in T2D. **b** An overview of the reactions that involve the identified metabolites. **c** The genes connected to each metabolite gene-set, together with their individual significance and fold change direction. Significantly differentially expressed genes (*q* < 0.05) in T2D versus controls are marked in *bold* and *color*. *Ceramide (Cer), glucosylceramide (GluCer), lactosylceramide (LacCer), galactosylceramide (GalCer), digalactosylceramide (DiGalCer), UDP-galactose (UDP-Gal), UDP-glucose (UDP-Glu)*

As both T2D and obesity are conditions associated with altered metabolism it was of interest to investigate whether any transcriptional changes related to metabolism occurred. GSA of metabolic pathways did not, however, identify any significant pathways. To not be constrained by classic metabolic pathway definitions, a separate analysis was run, identifying so called reporter metabolites [38]. These are metabolite gene-sets that are extracted from the topology of a myocyte genome-scale metabolic network [29]. The tool Kiwi [31] was used to visualize the significant metabolites and their interactions and connection in the metabolic network (Fig. 4a). A connected subnetwork of metabolites was identified, involving a specific part of sphingolipid metabolism (Fig. 4b). This subnetwork was affected by a general transcriptional up-regulation in T2D versus controls, even though individual genes showed mixed directions of change. The heatmap in Fig. 4c shows all connected genes underlying the identified network. Even though the significantly differentially expressed genes are highlighted, all connected genes contribute to the metabolite gene-set scores, regardless of an arbitrary cutoff, which is one of the benefits of GSA.

We also performed qPCR on ten selected genes involved in pathways and processes identified by the functional characterization of the RNA-seq expression data, using material from an additional set of in vitro myocytes from eight T2D and eight healthy males at baseline, i.e., without insulin stimulation (Additional file 3: Table S2) [39]. Due to high variance in these data, only one gene (*B3GNT5*, which encodes an enzyme catalyzing the first step of conversion of lactosylceramide into more complex glycosphingolipids) was found significantly differentially expressed between the T2D and control groups, with a fold change consistent with the results from the RNA-seq data analysis (Additional file 11: Figure S7).

### Characterizing differences between T2D and OB

Even though the results point to similar and consistent transcriptional responses associated with T2D and obesity, a small number of genes were differentially expressed between T2D and OB. A majority of these were also significant in one of the groups compared to controls (representing changes driven specifically by either T2D or OB), whereas a few genes were regulated in different directions in the two groups compared to controls (Additional file 12: Figure S8).

GO term and hallmark GSA results (for T2D versus OB) coincided with gene-sets affected in the comparison of the three groups with controls (Additional file 9: Figure S5; Additional file 10: Figure S6). This implies that most of the differences between T2D and OB reflect differences in the extent of regulation of the processes and functions associated with the similar transcriptional profile that we identified for T2D, OB, and T2D&OB. As an example of this, myogenesis, which was found down-regulated in all three groups compared to controls, was also down-regulated in T2D versus OB, showing that myogenesis was indeed down-regulated in both T2D and OB, but more pronounced in T2D.

## Discussion

**Fig. 5:**
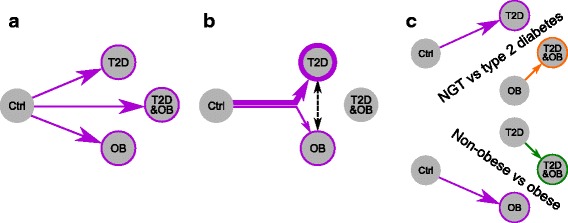
Summary of the transcriptional patterns across the four subject groups. **a** The T2D, OB, and T2D&OB groups display similar transcriptional changes compared with the controls. **b** The expression differences between T2D and OB mainly reflect differences in the extent of the similar changes from controls (see also Additional file 12: Figure S8). **c** The detected differences between T2D and NGT are dependent on the weight levels of the subjects. The difference between T2D and NGT in non-obese subjects is larger than for obese subjects. Similar observations were made for the obesity factor

It is well known that obesity is a risk factor for T2D and that these conditions typically appear together. Even so, it was remarkable that a myocyte derived from an obese person with no signs of T2D and a myocyte derived from a T2D but non-obese person converged on the same inherent transcriptional patterns, pointing to the myocytes of these subjects having very similar phenotypes (Fig. 5a). The few differences between the T2D and OB groups primarily reflected differences in the extent of these similar changes from controls (Fig. 5b). The lack of major differences could be due to reduced statistical power arising from a small number of biological replicates, and, by increasing the sample size, more distinct differences could potentially be identified. Nevertheless, more than 200 genes were still significantly differentially expressed in T2D versus OB, indicating that the similarities are not solely due to reduced statistical power. We also found that the clear distinction between NGT and T2D in the non-obese case was reduced in the obese case, indicating that obesity promotes an inherent phenotype similar to that of T2D, perhaps providing a foundation for developing the disease under certain conditions (Fig. 5c). It is important, however, to point out that obese subjects (both NGT and T2D) are hyperinsulinemic, which partly could explain the observed smaller differences. The effect of T2D on non-obese subjects (T2D versus controls) is more pronounced, and thereby different, than the effect of T2D on obese subjects (T2D&OB versus OB). This observation is important to take into account when conducting research or comparing results between studies that investigate the effect of, e.g., T2D on subject groups with different BMI levels.

We found a candidate epigenetic histone mark, H3K27me3, behind the inherent transcriptional landscape of myocytes derived from obese and T2D subjects. H3K27me3 has been reported to be associated with transcriptional repression [40–42]. A portion of the H3K27me3 gene-sets were specifically enriched by down-regulated genes and it is therefore possible that there is an increase of the H3K27me3 mark in myocytes from T2D, OB, and T2D&OB subjects. A previous study in adipocytes showed that a large number of genes can be influenced by genome-wide histone methylation differences in connection with T2D and overweight [43]. Nevertheless, as our data indicated a mix of both up- and down-regulation of H3K27me3 genes, it is plausible that specific changes in H3K27me3 levels take place in different genomic locations.

H3K27me3 is associated with transcriptional repression of genes involved in development and differentiation [40, 41] and the H3K27me3 mark is removed during myogenesis [44]. Consistently, our analyses revealed down-regulation of genes involved in myogenesis, muscle development, and muscle contraction, function, and structure. There was also a significant down-regulation of myogenic marker genes in the T2D group. It thus seems like part of the inherent transcriptional profile associated with both T2D and obesity involves down-regulation of genes involved in muscle development, mediated through differential H3K27 methylation compared to controls.

Intriguingly, we observed up-regulation, in all three groups, of genes involved in the function of the extracellular matrix (ECM), represented by the hallmark EMT gene-set, and several GO terms, including extracellular matrix, heparin binding, collagen binding, glycosaminoglycan binding, and integrin binding. ECM is important in muscle maintenance and regulation of muscle development and growth and is essential for myotube formation [45, 46]. A majority of the components of the ECM are produced by fibroblasts [47], but both muscle precursor cells (satellite cells) and multinucleated myofibers have been shown to contribute to the production of ECM [48–50]. Perhaps the up-regulation of genes involved in the ECM in combination with down-regulation of myogenesis and muscle function is a compensating mechanism, although this needs to be further investigated.

Sphingolipids, a diverse group of metabolites with roles in cell structure (including the ECM) and signaling, have previously been implicated in T2D and obesity [51, 52]. Increased sphingolipid production, plasma glycosphingolipid levels, and muscle ceramide levels, in association with T2D, have been reported in the literature before [53–55]. Inhibition of glucosylceramide synthase, catalyzing the production of glucosylceramide from ceramide (Fig. 3d), and thus reducing the production of a number of downstream glycosphingolipids, has been shown to result in improved insulin sensitivity [56]. Other studies have also reported a correlation between ceramide levels in muscle and reduced insulin sensitivity [57–59]. Here we found that the metabolism connecting ceramide and glycosphingolipids was transcriptionally up-regulated in myocytes originating from T2D subjects but kept in an identical in vitro environment as myocytes originating from control subjects. This provides evidence for the presence of inherent regulation of this part of metabolism, independent of circulating levels of, e.g., insulin or sphingolipids, corroborating the importance of sphingolipids in the pathogenesis of T2D in skeletal muscle.

Plasma ceramides have also been shown to correlate with levels of the inflammatory cytokines TNF-alpha and IL6 [54, 60] and have been suggested to be the mediator behind TNF-alpha-induced insulin resistance [61]. There is thus evidence of a link between sphingolipids, insulin resistance, and inflammation. Indeed, chronic low-grade inflammation and activation of the immune system have been shown to be involved in obesity and the pathogenesis of T2D [62, 63]. The role of muscle inflammation in connection to T2D is not fully understood. Macrophage infiltration has been observed in skeletal muscle of obese mice [64] and increased inflammatory macrophage gene expression was seen in skeletal muscle of T2D human patients [65]. In line with this, our analysis of GO term and hallmark gene-sets identified up-regulation and unspecific regulation of immune and inflammatory responses in the T2D, OB, and T2D&OB groups. It is interesting that the in vitro myocytes, which are not in contact with any macrophages, inherently expressed immune- and inflammation-related genes, solely as a consequence of having originated from muscle precursor cells from subjects with T2D or obesity.

## Conclusions

Our study enabled a systematic characterization of the individual effects of T2D and obesity on skeletal myocytes. In particular, by using the in vitro myocyte model we were able to identify several properties that were inherent to myocytes originating from T2D and obese subjects, distinctive from control myocytes cultured in the same environment. Markedly similar transcriptional profiles were observed in association with both T2D and obesity, perhaps as a result of epigenetic modifications inherited from the muscle precursor cells, reflecting a down-regulation of myogenesis and muscle function and up-regulation of genes involved in inflammation and ECM. Using a network-based approach, independent of classic metabolic pathway definitions, we were also able to identify up-regulation of a metabolite subnetwork involved in sphingolipid metabolism. These changes were inherently present as a molecular memory of the in vivo condition, without the influence from a diabetic or obese extracellular environment, highlighting their importance in the progression and treatment of T2D.

## Additional files

Additional file 1: Supplementary materials and methods. (PDF 348 kb)

Additional file 2: Table S1. Differential expression results (adjusted *p* values and fold changes) and gene annotation. (XLSX 5696 kb)

Additional file 3: Table S2. Clinical characteristics of muscle stem cell donors used for qPCR analysis. (PDF 225 kb)

Additional file 4: Table S3. Primer sequences for qPCR. (PDF 309 kb)

Additional file 5: Figure S1. Comparison of different linear models. (PDF 109 kb)

Additional file 6: Figure S2. Volcano plots and pairwise correlation between fold changes of the T2D, OB, and T2D&OB groups compared to controls. (PDF 2801 kb)

Additional file 7: Figure S3. Analysis of expressed quantitative trait loci. (PDF 105 kb)

Additional file 8: Figure S4. ENCODE histone modification gene-set analysis results. (PDF 91 kb)

Additional file 9: Figure S5. Hallmark gene-set analysis results. (PDF 46 kb)

Additional file 10: Figure S6. GO-term gene-set analysis results. (PDF 1195 kb)

Additional file 11: Figure S7. Quantitative PCR results. (PDF 64 kb)

Additional file 12: Figure S8. Transcriptional differences between T2D and OB. (PDF 132 kb)

## Abbreviations

BMI: Body mass index
ECM: Extracellular matrix
EMT: Epithelial–mesenchymal transition
FDR: False discovery rate
GO: Gene Ontology
GSA: Gene-set analysis
NGT: Normal glucose tolerance
OB: Obese
T2D: Type 2 diabetes.

## References

[1] Das SK, Elbein SC (2006). The genetic basis of type 2 diabetes. Cellscience.

[2] Scully T (2012). Diabetes in numbers. Nature.

[3] Doria A, Patti M-E, Kahn CR (2008). The emerging genetic architecture of type 2 diabetes. Cell Metab.

[4] Mokdad AH, Ford ES, Bowman BA, Dietz WH, Vinicor F, Bales VS, Marks JS (2003). Prevalence of obesity, diabetes, and obesity-related health risk factors, 2001. JAMA.

[5] Arner P, Pollare T, Lithell H (1991). Different aetiologies of Type 2 (non-insulin-dependent) diabetes mellitus in obese and non-obese subjects. Diabetologia.

[6] Stump CS, Henriksen EJ, Wei Y, Sowers JR (2006). The metabolic syndrome: role of skeletal muscle metabolism. Ann Med.

[7] Björnholm M, Zierath JR (2005). Insulin signal transduction in human skeletal muscle: identifying the defects in type II diabetes. Biochem Soc Trans.

[8] DeFronzo RA, Tripathy D (2009). Skeletal muscle insulin resistance is the primary defect in type 2 diabetes. Diabetes Care..

[9] Bouzakri K, Roques M, Gual P, Espinosa S, Guebre-Egziabher F, Riou J-P, Laville M, Le Marchand-Brustel Y, Tanti J-F, Vidal H (2003). Reduced activation of phosphatidylinositol-3 kinase and increased serine 636 phosphorylation of insulin receptor substrate-1 in primary culture of skeletal muscle cells from patients with type 2 diabetes. Diabetes.

[10] Broholm C, Brandt C, Schultz NS, Nielsen AR, Pedersen BK, Scheele C (2012). Deficient leukemia inhibitory factor signaling in muscle precursor cells from patients with type 2 diabetes. Am J Physiol Endocrinol Metab.

[11] Green CJ, Pedersen M, Pedersen BK, Scheele C (2011). Elevated NF-κB activation is conserved in human myocytes cultured from obese type 2 diabetic patients and attenuated by AMP-activated protein kinase. Diabetes.

[12] Scheele C, Nielsen S, Kelly M, Broholm C, Nielsen AR, Taudorf S, Pedersen M, Fischer CP, Pedersen BK (2012). Satellite cells derived from obese humans with type 2 diabetes and differentiated into myocytes in vitro exhibit abnormal response to IL-6. PLoS One.

[13] Gaster M, Petersen I, Højlund K, Poulsen P, Beck-Nielsen H (2002). The diabetic phenotype is conserved in myotubes established from diabetic subjects. Diabetes.

[14] Thorburn AW, Gumbiner B, Bulacan F, Brechtel G, Henry RR (1991). Multiple defects in muscle glycogen synthase activity contribute to reduced glycogen synthesis in non-insulin dependent diabetes mellitus. J Clin Investig.

[15] Pedersen M, Pedersen KK, Bruunsgaard H, Krabbe KS, Thomsen C, Færch K, Pedersen BK, Mortensen EL (2012). Cognitive functions in middle aged individuals are related to metabolic disturbances and aerobic capacity: a cross-sectional study. PLoS One.

[16] Bergström J (1975). Percutaneous needle biopsy of skeletal muscle in physiological and clinical research. Scand J Clin Lab Inv.

[17] Dobin A, Davis CA, Schlesinger F, Drenkow J, Zaleski C, Jha S, Batut P, Chaisson M, Gingeras TR (2013). STAR: ultrafast universal RNA-seq aligner. Bioinformatics.

[18] Li H, Handsaker B, Wysoker A, Fennell T, Ruan J, Homer N, Marth G, Abecasis G, Durbin R, Subgroup GPDP (2009). The Sequence Alignment/Map format and SAMtools. Bioinformatics.

[19] Anders S, Pyl PT, Huber W (2014). HTSeq—a Python framework to work with high-throughput sequencing data. Bioinformatics.

[20] Huber W, Carey VJ, Gentleman R, Anders S, Carlson M, Carvalho BS, Bravo HC, Davis S, Gatto L, Girke T (2015). Orchestrating high-throughput genomic analysis with Bioconductor. Nat Methods.

[21] Ritchie ME, Phipson B, Wu D, Hu Y, Law CW, Shi W, Smyth GK (2015). limma powers differential expression analyses for RNA-sequencing and microarray studies. Nucleic Acids Res.

[22] Robinson M, Oshlack A (2010). A scaling normalization method for differential expression analysis of RNA-seq data. Genome Biol.

[23] Robinson MD, McCarthy DJ, Smyth GK (2010). edgeR: a Bioconductor package for differential expression analysis of digital gene expression data. Bioinformatics.

[24] Law C, Chen Y, Shi W, Smyth G (2014). voom: precision weights unlock linear model analysis tools for RNA-seq read counts. Genome Biol.

[25] Smyth GK, Michaud J, Scott HS (2005). Use of within-array replicate spots for assessing differential expression in microarray experiments. Bioinformatics.

[26] Smyth GK. Linear models and empirical bayes methods for assessing differential expression in microarray experiments. Stat Appl Genet Mol Biol. 2004;3(1):3. 10.2202/1544-6115.1027 16646809

[27] Benjamini Y, Hochberg Y (1995). Controlling the false discovery rate: a practical and powerful approach to multiple testing. J R Stat Soc Ser B (Methodological).

[28] Väremo L, Nielsen J, Nookaew I (2013). Enriching the gene set analysis of genome-wide data by incorporating directionality of gene expression and combining statistical hypotheses and methods. Nucleic Acids Res.

[29] Väremo L, Scheele C, Broholm C, Mardinoglu A, Kampf C, Asplund A, Nookaew I, Uhlén M, Pedersen Bente K, Nielsen J (2015). Proteome- and transcriptome-driven reconstruction of the human myocyte metabolic network and its use for identification of markers for diabetes. Cell Rep.

[30] Oliveira AP, Patil KR, Nielsen J (2008). Architecture of transcriptional regulatory circuits is knitted over the topology of bio-molecular interaction networks. BMC Syst Biol..

[31] Väremo L, Gatto F, Nielsen J (2014). Kiwi: a tool for integration and visualization of network topology and gene-set analysis. BMC Bioinf..

[32] Garcia-Albornoz M, Thankaswamy-Kosalai S, Nilsson A, Varemo L, Nookaew I, Nielsen J (2014). BioMet Toolbox 2.0: genome-wide analysis of metabolism and omics data. Nucleic Acids Res.

[33] Lonsdale J, Thomas J, Salvatore M, Phillips R, Lo E, Shad S, Hasz R, Walters G, Garcia F, Young N (2013). The Genotype-Tissue Expression (GTEx) project. Nat Genet.

[34] Lan F, Bayliss PE, Rinn JL, Whetstine JR, Wang JK, Chen S, Iwase S, Alpatov R, Issaeva I, Canaani E (2007). A histone H3 lysine 27 demethylase regulates animal posterior development. Nature.

[35] Kuzmichev A, Nishioka K, Erdjument-Bromage H, Tempst P, Reinberg D (2002). Histone methyltransferase activity associated with a human multiprotein complex containing the Enhancer of Zeste protein. Genes Dev.

[36] Liberzon A, Birger C, Thorvaldsdóttir H, Ghandi M, Mesirov Jill P, Tamayo P (2015). The Molecular Signatures Database Hallmark Gene Set Collection. Cell Systems.

[37] Ashburner M, Ball CA, Blake JA, Botstein D, Butler H, Cherry JM, Davis AP, Dolinski K, Dwight SS, Eppig JT (2000). Gene Ontology: tool for the unification of biology. Nat Genet.

[38] Patil KR, Nielsen J (2005). Uncovering transcriptional regulation of metabolism by using metabolic network topology. Proc Natl Acad Sci U S A.

[39] Henriksen TI, Davidsen PK, Pedersen M, Schultz HS, Hansen NS, Larsen TJ, Vaag A, Pedersen BK, Nielsen S, Scheele C (2017). Dysregulation of a novel miR-23b/27b-p53 axis impairs muscle stem cell differentiation of humans with type 2 diabetes. Mol Metab.

[40] Boyer LA, Plath K, Zeitlinger J, Brambrink T, Medeiros LA, Lee TI, Levine SS, Wernig M, Tajonar A, Ray MK (2006). Polycomb complexes repress developmental regulators in murine embryonic stem cells. Nature.

[41] Lee TI, Jenner RG, Boyer LA, Guenther MG, Levine SS, Kumar RM, Chevalier B, Johnstone SE, Cole MF, Isono K-I (2006). Control of developmental regulators by Polycomb in human embryonic stem cells. Cell.

[42] Barski A, Cuddapah S, Cui K, Roh T-Y, Schones DE, Wang Z, Wei G, Chepelev I, Zhao K (2007). High-resolution profiling of histone methylations in the human genome. Cell.

[43] Jufvas A, Sjodin S, Lundqvist K, Amin R, Vener A, Stralfors P (2013). Global differences in specific histone H3 methylation are associated with overweight and type 2 diabetes. Clin Epigenet.

[44] Seenundun S, Rampalli S, Liu QC, Aziz A, Palii C, Hong S, Blais A, Brand M, Ge K, Dilworth FJ (2010). UTX mediates demethylation of H3K27me3 at muscle‐specific genes during myogenesis. EMBO J.

[45] Velleman SG, Shin J, Li X, Song Y (2012). Review: The skeletal muscle extracellular matrix: Possible roles in the regulation of muscle development and growth. Can J Anim Sci.

[46] Melo F, Carey DJ, Brandan E (1996). Extracellular matrix is required for skeletal muscle differentiation but not myogenin expression. J Cell Biochem.

[47] Gatchalian CL, Schachner M, Sanes JR (1989). Fibroblasts that proliferate near denervated synaptic sites in skeletal muscle synthesize the adhesive molecules tenascin(J1), N-CAM, fibronectin, and a heparan sulfate proteoglycan. J Cell Biol.

[48] Guérin CW, Holland PC (1995). Synthesis and secretion of matrix-degrading metalloproteases by human skeletal muscle satellite cells. Dev Dyn.

[49] Gillies AR, Lieber RL (2011). Structure and function of the skeletal muscle extracellular matrix. Muscle Nerve.

[50] Beach RL, Rao JS, Festoff BW (1985). Extracellular-matrix synthesis by skeletal muscle in culture. Major secreted collagenous proteins of clonal myoblasts. Biochem J.

[51] Russo SB, Ross JS, Cowart LA (2013). Sphingolipids in obesity, type 2 diabetes, and metabolic disease. Handb Exp Pharmacol..

[52] Gault CR, Obeid LM, Hannun YA (2010). An overview of sphingolipid metabolism: from synthesis to breakdown. Adv Exp Med Biol..

[53] Summers SA, Nelson DH (2005). A role for sphingolipids in producing the common features of type 2 diabetes, metabolic syndrome X, and Cushing’s syndrome. Diabetes.

[54] Haus JM, Kashyap SR, Kasumov T, Zhang R, Kelly KR, DeFronzo RA, Kirwan JP (2009). Plasma ceramides are elevated in obese subjects with type 2 diabetes and correlate with the severity of insulin resistance. Diabetes.

[55] Adams JM, Pratipanawatr T, Berria R, Wang E, DeFronzo RA, Sullards MC, Mandarino LJ (2004). Ceramide content is increased in skeletal muscle from obese insulin-resistant humans. Diabetes.

[56] Zhao H, Przybylska M, Wu IH, Zhang J, Siegel C, Komarnitsky S, Yew NS, Cheng SH (2007). Inhibiting glycosphingolipid synthesis improves glycemic control and insulin sensitivity in animal models of type 2 diabetes. Diabetes.

[57] Amati F, Dubé JJ, Alvarez-Carnero E, Edreira MM, Chomentowski P, Coen PM, Switzer GE, Bickel PE, Stefanovic-Racic M, Toledo FGS (2011). Skeletal muscle triglycerides, diacylglycerols, and ceramides in insulin resistance: another paradox in endurance-trained athletes?. Diabetes.

[58] Straczkowski M, Kowalska I, Nikolajuk A, Dzienis-Straczkowska S, Kinalska I, Baranowski M, Zendzian-Piotrowska M, Brzezinska Z, Gorski J (2004). Relationship between insulin sensitivity and sphingomyelin signaling pathway in human skeletal muscle. Diabetes.

[59] Straczkowski M, Kowalska I, Baranowski M, Nikolajuk A, Otziomek E, Zabielski P, Adamska A, Blachnio A, Gorski J, Gorska M (2007). Increased skeletal muscle ceramide level in men at risk of developing type 2 diabetes. Diabetologia.

[60] de Mello VDF, Lankinen M, Schwab U, Kolehmainen M, Lehto S, Seppänen-Laakso T, Orešič M, Pulkkinen L, Uusitupa M, Erkkilä AT (2009). Link between plasma ceramides, inflammation and insulin resistance: association with serum IL-6 concentration in patients with coronary heart disease. Diabetologia.

[61] Teruel T, Hernandez R, Lorenzo M (2001). Ceramide mediates insulin resistance by tumor necrosis factor-α in brown adipocytes by maintaining Akt in an inactive dephosphorylated state. Diabetes.

[62] Donath MY, Shoelson SE (2011). Type 2 diabetes as an inflammatory disease. Nat Rev Immunol.

[63] Esser N, Legrand-Poels S, Piette J, Scheen AJ, Paquot N (2014). Inflammation as a link between obesity, metabolic syndrome and type 2 diabetes. Diabetes Res Clin Pr.

[64] Weisberg SP, McCann D, Desai M, Rosenbaum M, Leibel RL, Ferrante AW (2003). Obesity is associated with macrophage accumulation in adipose tissue. J Clin Invest.

[65] Fink LN, Oberbach A, Costford SR, Chan KL, Sams A, Blüher M, Klip A (2013). Expression of anti-inflammatory macrophage genes within skeletal muscle correlates with insulin sensitivity in human obesity and type 2 diabetes. Diabetologia.

