# Supplementary material for: Type 2 diabetes and obesity induce similar transcriptional reprogramming in human myocytes
Source: Genome Med. 2017 May 25;9:47. doi: 10.1186/s13073-017-0432-2 (PMC5444103; doi:10.1186/s13073-017-0432-2)
Supplement: Supplementary file 1 — Supplementary materials and methods. (PDF 348 kb) [file 13073_2017_432_MOESM1_ESM.pdf]

SUPPLEMENTARY MATERIAL AND METHODS

Leif Väremo, Tora Ida Henriksen, Camilla Scheele, Christa Broholm, Maria Pedersen, Mathias Uhlén, Bente Klarlund Pedersen, Jens Nielsen

Subjects and phenotype measurements

Study participants were recruited by advertising in a local newspaper. Exclusion criteria were treatment with insulin, recent or ongoing infection, history of malignant disease, treatment with anti-inflammatory drugs, use of thiazolidinedione-based medications within 3 months, or unstable body weight (±10%) for the past year. Participation also required normal results from the physical examination; electrocardiogram; blood tests for renal function (creatinine), hepatic function (alanine aminotransferase), and thyroid function (thyroid-stimulating hormone); hemoglobin; white blood cell counts; and electrolytes and urinalysis. Using a cross sectional case-control design, participants (n = 24) were divided into four groups based on whether the participants had normal glucose tolerance or T2D according to the result of an oral glucose tolerance test (OGTT). The WHO diagnostic criteria for T2D diabetes were used (1). The participants were subsequently divided into two groups based on their BMI (over 30 versus below 30). All participants were given both oral and written information about the experimental procedures before giving their written informed consent.

Serum cholesterol was measured after an overnight fast, plasma glucose and serum insulin were measured before and 2 hours after a standard 75 g oral glucose tolerance test (OGTT) and a muscle biopsy was taken on a different test day. Homeostatic model assessment (HOMA) is a method used to quantify insulin resistance (HOMA-IR) (2). HOMA-IR values were calculated from fasting glucose and insulin blood concentrations using the HOMA2 Calculator 2.2.3 (Diabetes Trials Unit, University of Oxford, http://www.dtu.ox.ac.uk/homa). Patients with T2D were not allowed taking any antidiabetic medication for one week preceding the test days.

Culturing of myocytes and sampling of RNA

Muscle precursor cells (satellite cells) were isolated and cultured in growth media (HAM/F10 supplied with 20% Fetal Bovine Serum and 1% penicillin/streptomycin) and plated in 6-well plates. Upon 70-80% confluence, the media was changed to differentiation media 1 (DMEM 4.5 g/L glucose supplied with 10% FBS and 1% penicillin/streptomycin) for two-three days. When the myoblasts had lined up, the media was changed to differentiation media 2 (DMEM 4.5 g/L glucose supplied with 2% horse serum and 1% penicillin/streptomycin) thereby initiating fusion into myotubes. Media was changed every second day. Cultures were fully differentiated by day 5 of incubation with differentiation media 2, as determined by visual confirmation of myotube formation (>3 nuclei per myotube in ~70% of the cells). At day 5, media was changed to DMEM 1.0 g/L glucose without any supplements for two hours and the myotubes were subsequently stimulated with insulin (100nM). Cells were harvested before insulin stimulation, 30 minutes, one hour or two hours after insulin stimulation and total RNA was extracted using TRIzol® (Life Sciences) according to the manufacturer's instructions. Frozen samples were shipped to the sequencing facility where they were purified by poly-A enrichment using Illumina TruSeq RNA.

eQTL analysis

The NCBI GTex eQTL Browser (3) (http://www.ncbi.nlm.nih.gov/gtex/test/GTEX2) was used for acquiring eQTLs associated with obesity and T2D. The search parameter Phenotype traits was set to "Obesity", "Obesity (extreme)", and "Type 2 diabetes", and the Association Test Significance Filter was set to a p-value of 0.001. This resulted in 30 unique genes with expression changes connected to a total of 158 SNPs. Out of these, 7 genes were also significant in our data. For each SNP and each subject, the consensus nucleotide sequence was determined based on the RNA-seq alignment data, around each SNP location. First, for each position, the base with the highest frequency among the aligned reads was determined for each sample. In many cases this could not be determined as no sequences were aligned at this location. Next, as each subject is represented by four samples, at each position, the base was determined to be the most common one determined for the four samples. In case of a tie the base was left undetermined and set to N. In this way, it was possible to get a consensus nucleotide sequence for each subject, and thus possible to make base distributions (A, T, C, G, or N if undetermined) for each SNP, and flanking sequences, across the 24 subjects. These sequences were visualized using Jalview (4).

Differential expression analysis

To capture the main effects of T2D and obesity as well as their possible influence on each other, a full interaction model was used. Further on, factors were included to account for expression related to the time course after insulin stimulation (continuous factor), age (continuous factor), and sex (binary factor). The final model was formulated as follows (where T2D:OB denotes the interaction term):

Gene expression ~ Intercept + T2D + OB + T2D:OB + time + sex + age

Testing the T2D and OB coefficients is equivalent to assessing differential expression between the T2D group and controls and the OB group and controls, respectively. Testing the contrast T2D + OB + T2D:OB is equivalent to assessing differential expression between the T2D&OB group and controls. Finally, testing the contrast T2D - OB is equivalent of comparing the T2D group to the OB group.

Gene-set analysis

Histone modification gene-sets ("Epigenomics_Roadmap_HM_ChIP-seq" and "ENCODE_Histone_ Modifications_2015") were downloaded from Enrichr (http://amp.pharm.mssm.edu/Enrichr/) (5). Only gene-sets for skeletal muscle, cultured muscle satellite cells, and smooth muscle (Epigenomics) or human myotubes, human myoblasts, and mouse myocytes (ENCODE), were considered in the analysis. Hallmark gene-sets were downloaded from the Molecular Signatures Database v5.0 (http://software.broadinstitute.org/gsea/msigdb/collections.jsp) (6). GO-term gene-sets were acquired using the Bioconductor R package biomaRt (7) and filtered so that gene-sets with fewer than 10 and more than 300 genes were excluded. Pathway gene-sets were extracted from the myocyte metabolic network iMyocyte2419 (8).

Piano reports the gene-set results in several different classes, to capture the general direction of gene-level regulation. This information was condensed into a heatmap (Figure 2, 3A-B, S4A, S5, S6), as follows. If at least one "directional up"-class had average $q<0.001$ the gene-set was regarded up-regulated (red), and the reverse for down-regulation (blue). However, if a combination of "directional up" and "directional down"-classes were significant (average $q<0.001$) the gene-set was considered to have unspecific regulation (green). This was also the case if only the non-directional class was significant. If the non-directional class had average $q>0.001$ the gene-set was not considered significant (white).

Supplementary References

1. WHO and IDF. (2006) *Definition and diagnosis of diabetes mellitus and intermediate hyperglycaemia*, Geneva.
2. Matthews, D.R., Hosker, J.P., Rudenski, A.S., Naylor, B.A., Treacher, D.F. and Turner, R.C. (1985) Homeostasis model assessment: insulin resistance and β-cell function from fasting plasma glucose and insulin concentrations in man. *Diabetologia*, **28**, 412-419.
3. Lonsdale, J., Thomas, J., Salvatore, M., Phillips, R., Lo, E., Shad, S., Hasz, R., Walters, G., Garcia, F., Young, N. *et al.* (2013) The Genotype-Tissue Expression (GTEx) project. *Nat Genet*, **45**, 580-585.
4. Waterhouse, A.M., Procter, J.B., Martin, D.M.A., Clamp, M. and Barton, G.J. (2009) Jalview Version 2—a multiple sequence alignment editor and analysis workbench. *Bioinformatics*, **25**, 1189-1191.
5. Chen, E., Tan, C., Kou, Y., Duan, Q., Wang, Z., Meirelles, G., Clark, N. and Ma'ayan, A. (2013) Enrichr: interactive and collaborative HTML5 gene list enrichment analysis tool. *BMC Bioinf.*, **14**, 128.
6. Liberzon, A., Subramanian, A., Pinchback, R., Thorvaldsdóttir, H., Tamayo, P. and Mesirov, J.P. (2011) Molecular signatures database (MSigDB) 3.0. *Bioinformatics*, **27**, 1739-1740.
7. Durinck, S., Moreau, Y., Kasprzyk, A., Davis, S., De Moor, B., Brazma, A. and Huber, W. (2005) BioMart and Bioconductor: a powerful link between biological databases and microarray data analysis. *Bioinformatics*, **21**, 3439-3440.
8. Väremo, L., Scheele, C., Broholm, C., Mardinoglu, A., Kampf, C., Asplund, A., Nookaew, I., Uhlén, M., Pedersen, Bente K. and Nielsen, J. (2015) Proteome- and Transcriptome-Driven Reconstruction of the Human Myocyte Metabolic Network and Its Use for Identification of Markers for Diabetes. *Cell Reports*, **11**, 921-933.
