# Supplementary material for: Type 2 diabetes and obesity induce similar transcriptional reprogramming in human myocytes
Source: Genome Med. 2017 May 25;9:47. doi: 10.1186/s13073-017-0432-2 (PMC5444103; doi:10.1186/s13073-017-0432-2)
Supplement: Supplementary file 3 — Clinical characteristics of muscle stem cell donors used for qPCR analysis. (PDF 225 kb) [file 13073_2017_432_MOESM3_ESM.pdf]

Table S2. Clinical characteristics of muscle stem cell donors, used for qPCR analysis

	Healthy (n=8)	T2D (n=8)
**Age (years)**	58.0 (50-67)	58.9 (51-65)
**BMI (kg/m2)**	25.4±1.8	26.2±2.8
**Fasting glucose (mmol/L)**	5.6±0.6	11.3±4.4**
**OGTT 2-hour glucose (mmol/L)**	5.1±1.8	20.2±3.8****
**Fasting insulin (pmol/L)**	29.9±16.5	42.3±17.0
**OGTT 2-hour insulin (pmol/L)**	154.±125.9	152.6±46.1
**HOMA-IR**	1.2±0.7	3.9±3.1*
**VO2 max (L/min)**	3.0±0.6	2.3±0.8

Note that four of the 24 subjects which the RNA-seq analysis was based on, were also included here (one T2D/non-OB and three NGT/non-OB males). Data are means ± SE. BMI= body mass index; OGTT= oral glucose tolerance test. Glucose values are mmol/L, insulin values are pmol/L. Differences between groups were compared using student's unpaired t-test. * P<0.05, **P<0.001, **** P<0.0001.
