# Supplementary material for: Type 2 diabetes and obesity induce similar transcriptional reprogramming in human myocytes
Source: Genome Med. 2017 May 25;9:47. doi: 10.1186/s13073-017-0432-2 (PMC5444103; doi:10.1186/s13073-017-0432-2)
Supplement: Supplementary file 4 — Primer sequences for qPCR. (PDF 309 kb) [file 13073_2017_432_MOESM4_ESM.pdf]

Table S3. Primer sequences for qPCR

Gene	Forward primer	Reverse primer
TNN1	GGGTGACTGGAGGAAGAACG	AGCAAGCTGGCCTCTATTGT
CTST	TGACTACAAGTGGTTTGCCTTTT	TAGCCCCAACGTCTGTTCAT
MEF2C	CAGTGCAGGGAACGGGTATG	GCAGGTCGACATCCTCAGAC
AIRE	CCCAGGCTCTCAACTGAAGG	GTCTGAATCCCGTTCCCGAG
NFATC4	TCTTCCTTCCTCCTCCAGCC	GTCCAGTTCTTCCCCCAATCC
TNFSF4	TCTTCCCTCTACCCAGATTGTGA	CGATGTGATACCTGAAGAGCAG
DEGS1	CCGGGAGATCCTGGCAAAGT	CCCAACTGGGTGAGAACCAT
B3GNT5	CTTTAGCTCCGATGCGGGAA	ATTCCATGCCACCTCCAAGTC
ELOVL3	TCGAGGAGTATTGGGCAACC	AATGCCCCACATCCTCACTG
B4GALT5	CCGGCATAGTGAACACCTACC	TAAGCACTCCGAAGCACCTG
PPIA	ACGCCACCGCCGAGGAAAAC	TGCAAACAGCTCAAAGGAGACGC
