# Supplementary material for: Type 2 diabetes and obesity induce similar transcriptional reprogramming in human myocytes
Source: Genome Med. 2017 May 25;9:47. doi: 10.1186/s13073-017-0432-2 (PMC5444103; doi:10.1186/s13073-017-0432-2)
Supplement: Supplementary file 5 — Comparison of different linear models. (PDF 109 kb) [file 13073_2017_432_MOESM5_ESM.pdf]

Comparison of different linear models

1. Original model Intercept + T2D + OB + T2D:OB + time + sex + age

Number of significant ($q<0.05$) genes for each factor:

	T2D	OB	T2D:OB	time	sex	age
1. Original model	2073	1425	825	5600	364	107

2. Without duplicateCorrelation Intercept + T2D + OB + T2D:OB + time + sex + age

T2D	OB	T2D:OB	time	sex	age
7580	6718	5710	1860	3471	1605

3. Without gender and age Intercept + T2D + OB + T2D:OB + time

T2D	OB	T2D:OB	time
2332	1032	636	5539

4. 24 baseline samples only Intercept + T2D + OB + T2D:OB + sex + age

T2D	OB	T2D:OB	sex	age
0	1	0	32	0

Spearman correlation of the significance of the factors in the 4 different models

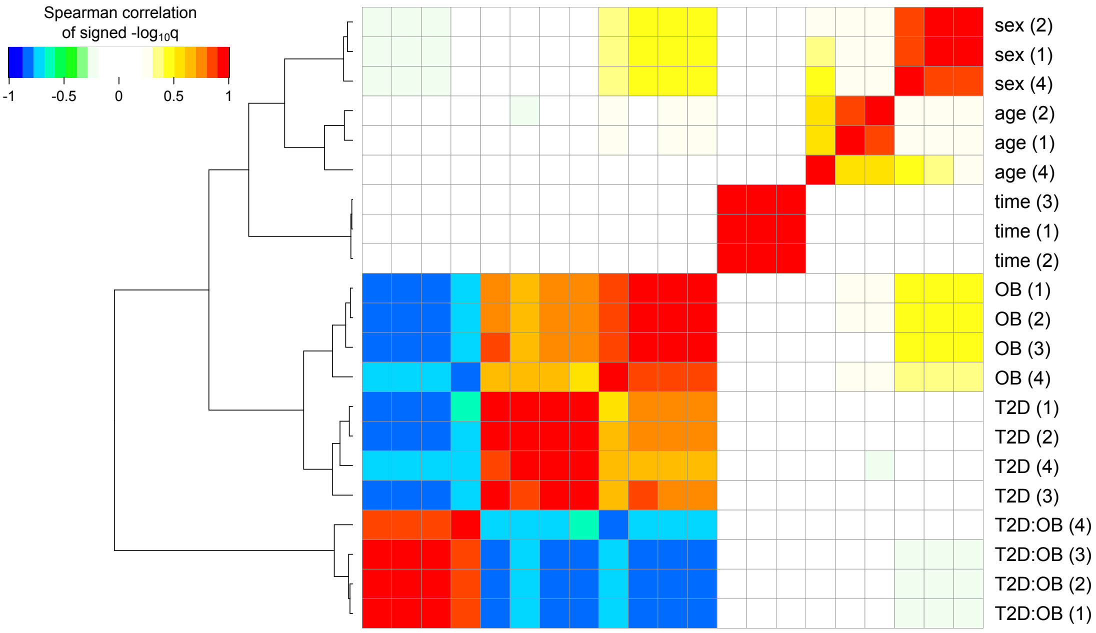

Testing interactions between insulin (time) and T2D and OB

Intercept + T2D + OB + T2D:OB + time + time:T2D + time:OB + time:T2D:OB + sex + age

T2D	OB	T2D:OB	time	time:T2D	time:OB	time:T2D:OB	sex	age
1627	884	346	778	0	0	0	318	98

Intercept + T2D + OB + T2D:OB + time + time:T2D + time:OB + time:T2D:OB

T2D	OB	T2D:OB	time	time:T2D	time:OB	time:T2D:OB
1815	535	231	768	0	0	0

Intercept + T2D + OB + T2D:OB + time + time:T2D + time:OB

T2D	OB	T2D:OB	time	time:T2D	time:OB
2178	809	604	1190	0	0

No significant interactions between insulin and T2D or OB!

Figure S1. Four different linear models are shown with varying factors included. The number of significant genes are shown for each factor. The handling of correlation of samples from the same subjects (original model) reduces the number of significant genes for most factors, compared to model 2. The inclusion or exclusion of sex and age does not drastically change the number of significant genes. The use of baseline (time = 0h) samples only does not give enough power to detect any significant genes. Nevertheless, gene-level q-values of the same factors across the different models have high correlation, as shown in the heatmap, meaning that the biological conclusions are robust to variations in the linear model structure. The inclusion of different combinations of interaction terms involving time (insulin) did not result in any significance, thus the effect of insulin on transcription in the four different groups can be explained by a single factor.
