# Supplementary material for: Type 2 diabetes and obesity induce similar transcriptional reprogramming in human myocytes
Source: Genome Med. 2017 May 25;9:47. doi: 10.1186/s13073-017-0432-2 (PMC5444103; doi:10.1186/s13073-017-0432-2)
Supplement: Supplementary file 6 — Volcano plots and pairwise correlation between fold changes of the T2D, OB, and T2D&OB groups compared to controls. (PDF 2801 kb) [file 13073_2017_432_MOESM6_ESM.pdf]

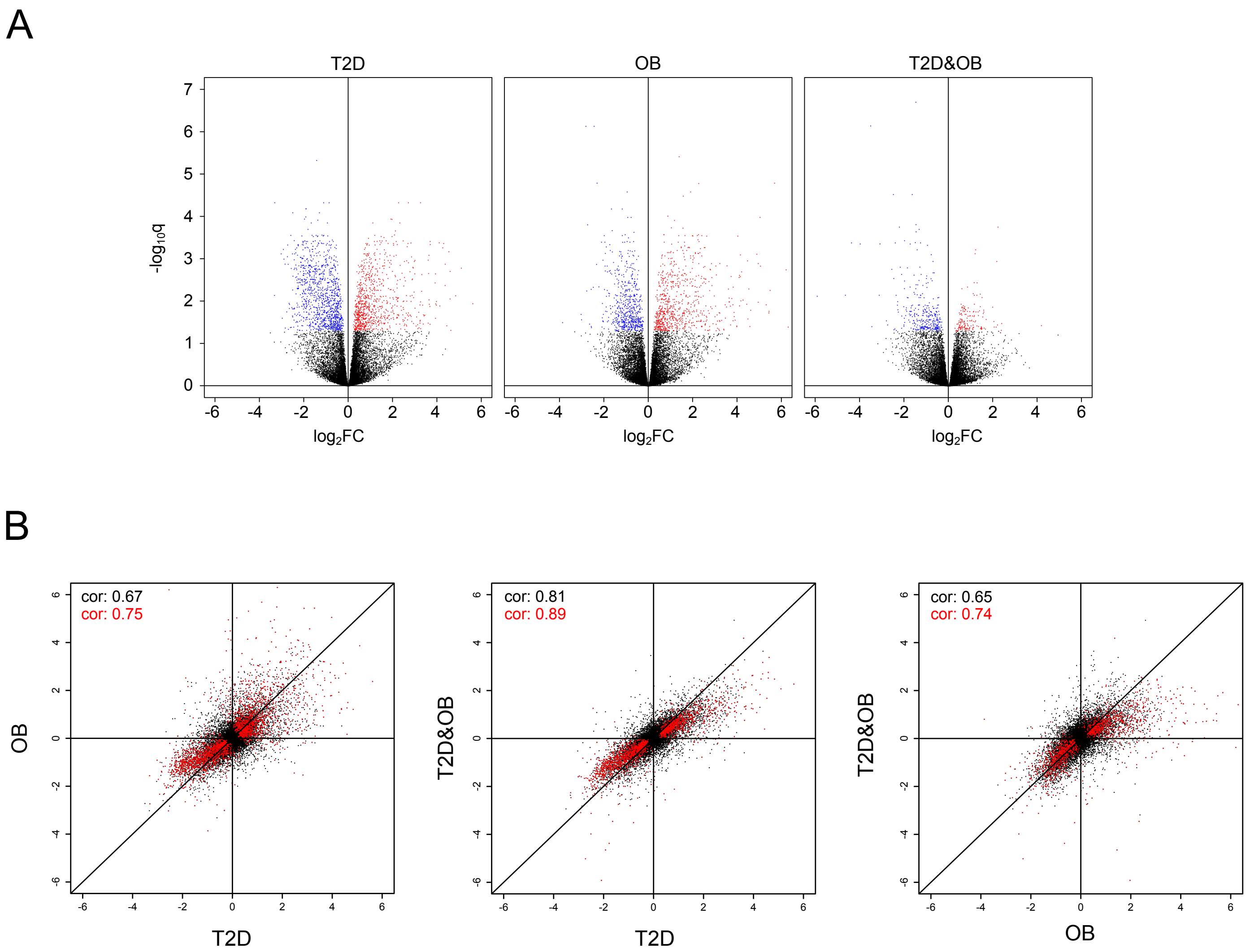

Figure S2. (A) Volcano plots showing gene fold changes and q-values for the three groups (T2D, OB, and T2D&OB) compared to controls. Significantly differentially expressed genes ($q<0.05$) are highlighted in blue and red. One extreme data point in the T2D&OB group (log2-fold change of -5 and q=7.9e-12) is not shown. **(B)** Scatterplots of log2-fold changes (vs controls). Pearson correlation is shown in black for all genes and in red for genes that were significant in at least one of the compared groups ($q<0.05$).
