# Supplementary material for: Type 2 diabetes and obesity induce similar transcriptional reprogramming in human myocytes
Source: Genome Med. 2017 May 25;9:47. doi: 10.1186/s13073-017-0432-2 (PMC5444103; doi:10.1186/s13073-017-0432-2)
Supplement: Supplementary file 7 — Analysis of expressed quantitative trait loci. (PDF 105 kb) [file 13073_2017_432_MOESM7_ESM.pdf]

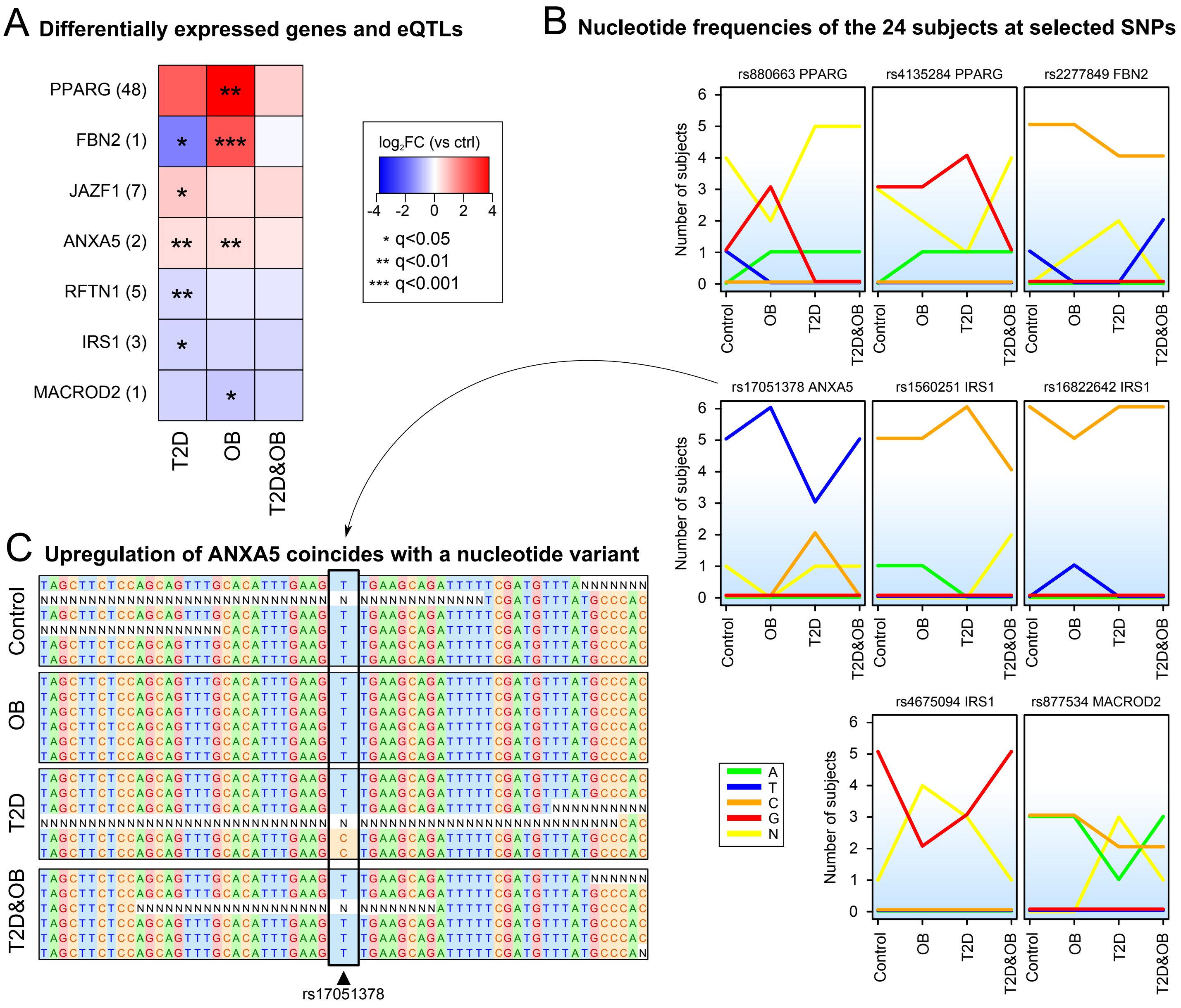

Figure S3. Potential influence on the transcriptional signatures from known eQTLs. **(A)** A heatmap showing fold changes of the genes that were both differentially expressed in at least one of the three groups (T2D, OB, or T2D&OB) and representing an eQTL associated with T2D or obesity. The number of SNPs associated with the expression of each gene are shown in parenthesis. **(B)** The nucleotide frequencies could be determined from the RNA-seq data (at least one group with sequences for at least 4 subjects) for 8 of the 67 SNPs. **(C)** Sequence details around one of the SNPs, associated with ANXA5, showing the higher incidence of the C nucleotide in T2D subjects, compared with the other groups. This example also illustrates the consistent flanking sequences across subjects and variation only detected in the actual SNP locus.
