# Supplementary material for: Type 2 diabetes and obesity induce similar transcriptional reprogramming in human myocytes
Source: Genome Med. 2017 May 25;9:47. doi: 10.1186/s13073-017-0432-2 (PMC5444103; doi:10.1186/s13073-017-0432-2)
Supplement: Supplementary file 8 — ENCODE histone modification gene-set analysis results. (PDF 91 kb) [file 13073_2017_432_MOESM8_ESM.pdf]

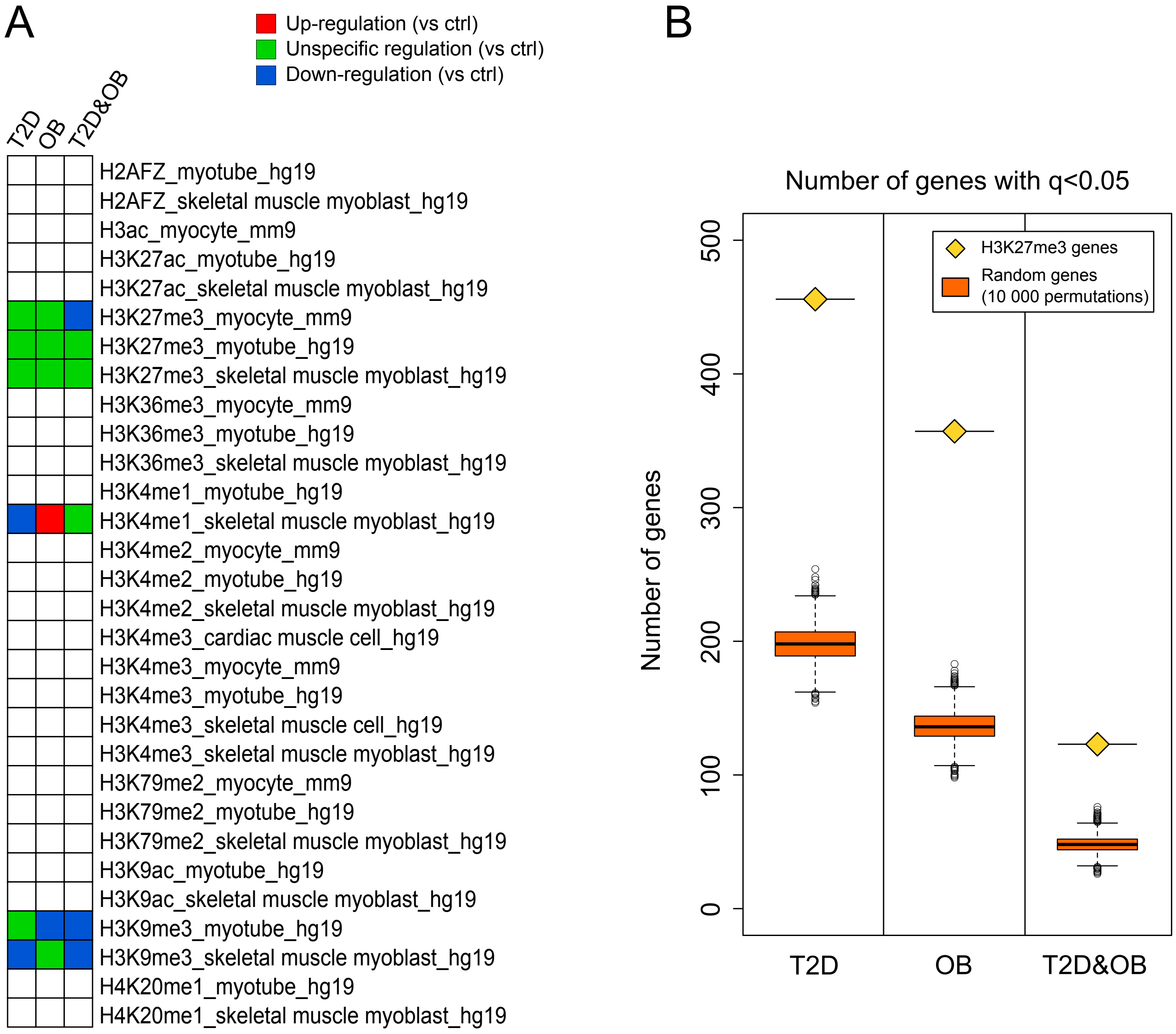

Figure S4. (A) A heatmap showing all investigated ENCODE histone modification gene-sets. Significant gene-sets are marked as either red (up-regulated), blue (down-regulated), or green (unspecifically regulated). **(B)** A plot showing the number of significantly differentially expressed H3K27me3 genes (i.e. belonging to one of the H3K27me3 gene-sets in Figure 2). These numbers are distinctively higher than the number of significant genes among randomly permuted genes (repeated 10,000 times).
