# Supplementary material for: Type 2 diabetes and obesity induce similar transcriptional reprogramming in human myocytes
Source: Genome Med. 2017 May 25;9:47. doi: 10.1186/s13073-017-0432-2 (PMC5444103; doi:10.1186/s13073-017-0432-2)
Supplement: Supplementary file 9 — Hallmark gene-set analysis results. (PDF 46 kb) [file 13073_2017_432_MOESM9_ESM.pdf]

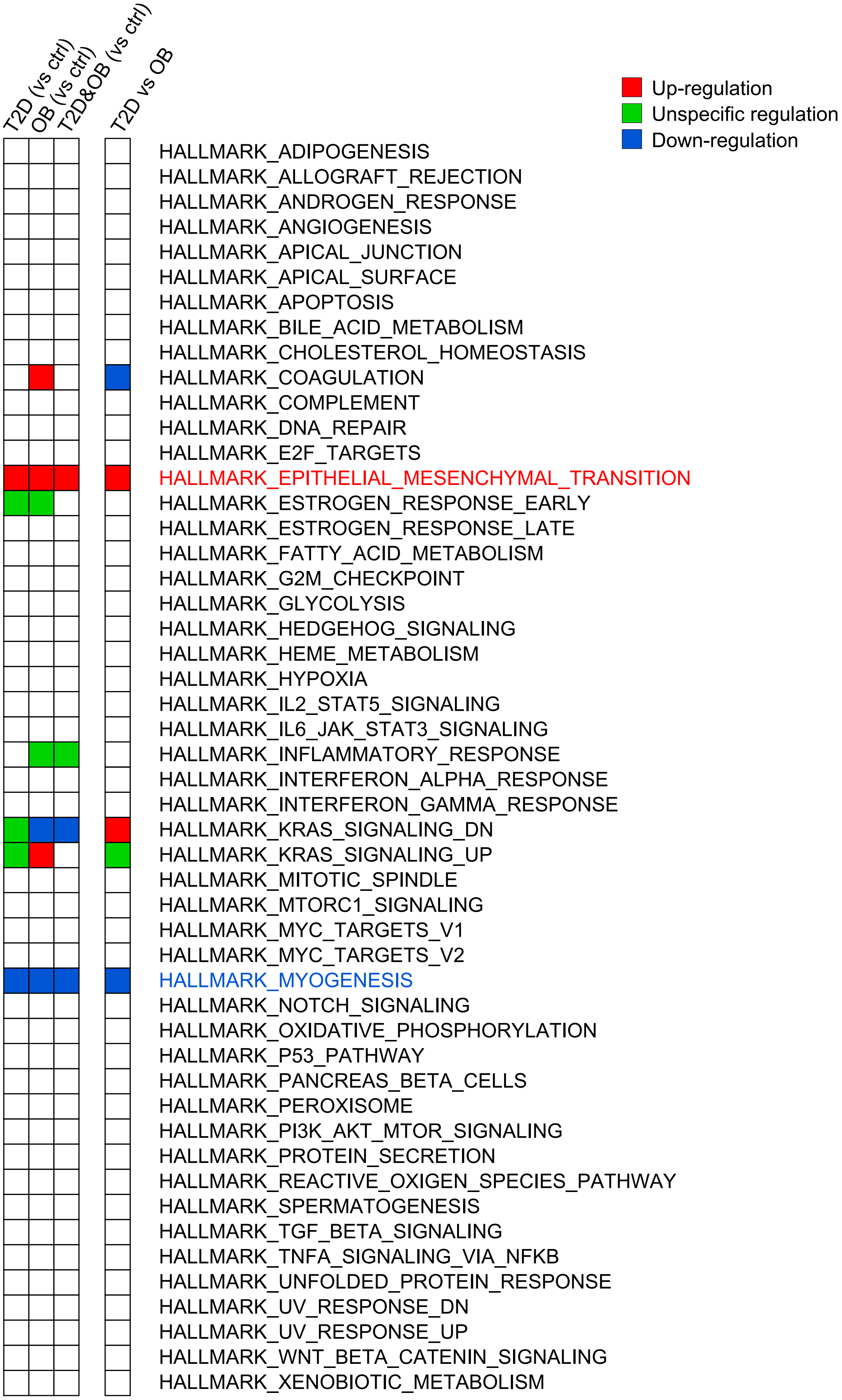

Figure S5. A heatmap showing all investigated hallmark gene-sets. Significant gene-sets are marked as either red (up-regulated), blue (down-regulated), or green (unspecifically regulated).
