# Supplementary material for: Type 2 diabetes and obesity induce similar transcriptional reprogramming in human myocytes
Source: Genome Med. 2017 May 25;9:47. doi: 10.1186/s13073-017-0432-2 (PMC5444103; doi:10.1186/s13073-017-0432-2)
Supplement: Supplementary file 10 — GO-term gene-set analysis results. (PDF 1195 kb) [file 13073_2017_432_MOESM10_ESM.pdf]

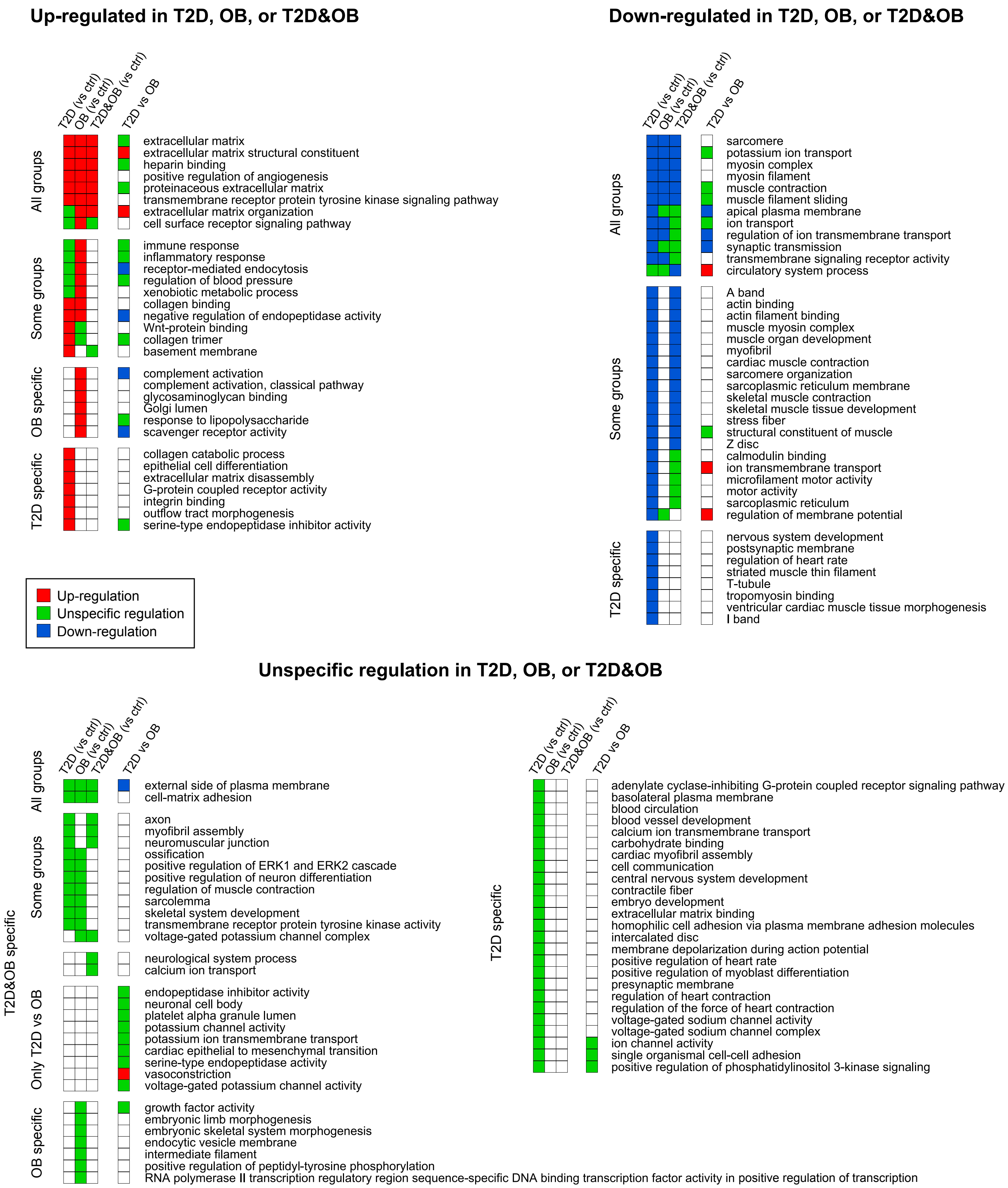

Figure S6. Heatmaps showing significant GO-term gene-sets. Significant gene-sets are marked as either red (up-regulated), blue (down-regulated), or green (unspecifically regulated).
