# Supplementary material for: Type 2 diabetes and obesity induce similar transcriptional reprogramming in human myocytes
Source: Genome Med. 2017 May 25;9:47. doi: 10.1186/s13073-017-0432-2 (PMC5444103; doi:10.1186/s13073-017-0432-2)
Supplement: Supplementary file 11 — Quantitative PCR results. (PDF 64 kb) [file 13073_2017_432_MOESM11_ESM.pdf]

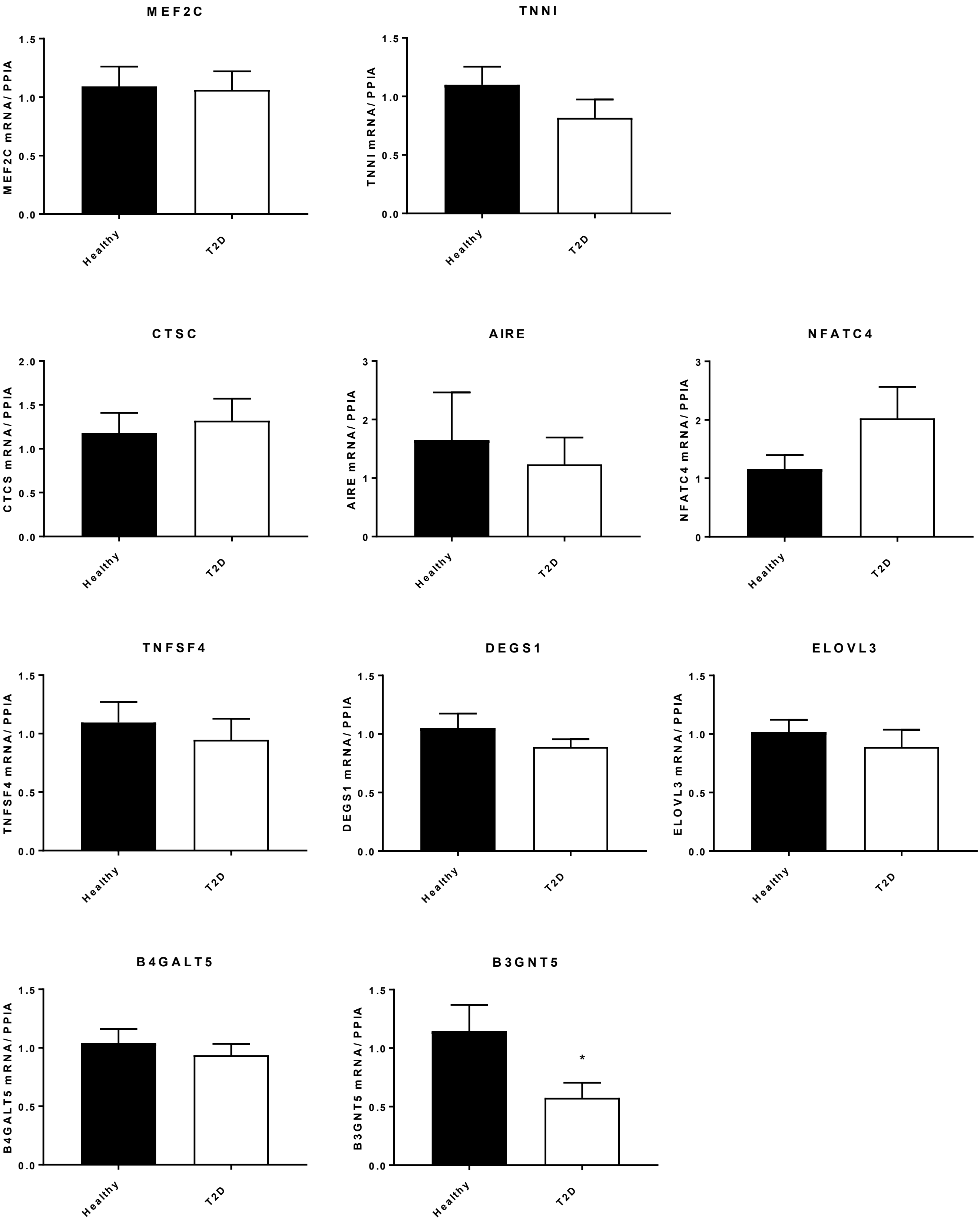

Figure S7. A few genes that were differentially expressed and related to sphingolipid/ceramide metabolism (DEGS1, B3GNT5, ELOVL3, B4GALT5), differentiation/development (TNNI1, MEF2C), and inflammatory/immune processes (CTSC, AIRE, NFATC4, TNFSF4) were selected for validation using qPCR. Due to high variability in the qPCR data, only one of the ten genes (B3GNT5, an enzyme catalyzing the first step of conversion of lactosylceramide into more complex glycosphingolipids) was found significantly differentially expressed (unpaired student's T-test, $P<0.05$) between the T2D and control group, with a fold-change consistent with the results from the RNA-seq data analysis.
