# Supplementary material for: Type 2 diabetes and obesity induce similar transcriptional reprogramming in human myocytes
Source: Genome Med. 2017 May 25;9:47. doi: 10.1186/s13073-017-0432-2 (PMC5444103; doi:10.1186/s13073-017-0432-2)
Supplement: Supplementary file 12 — Transcriptional differences between T2D and OB. (PDF 132 kb) [file 13073_2017_432_MOESM12_ESM.pdf]

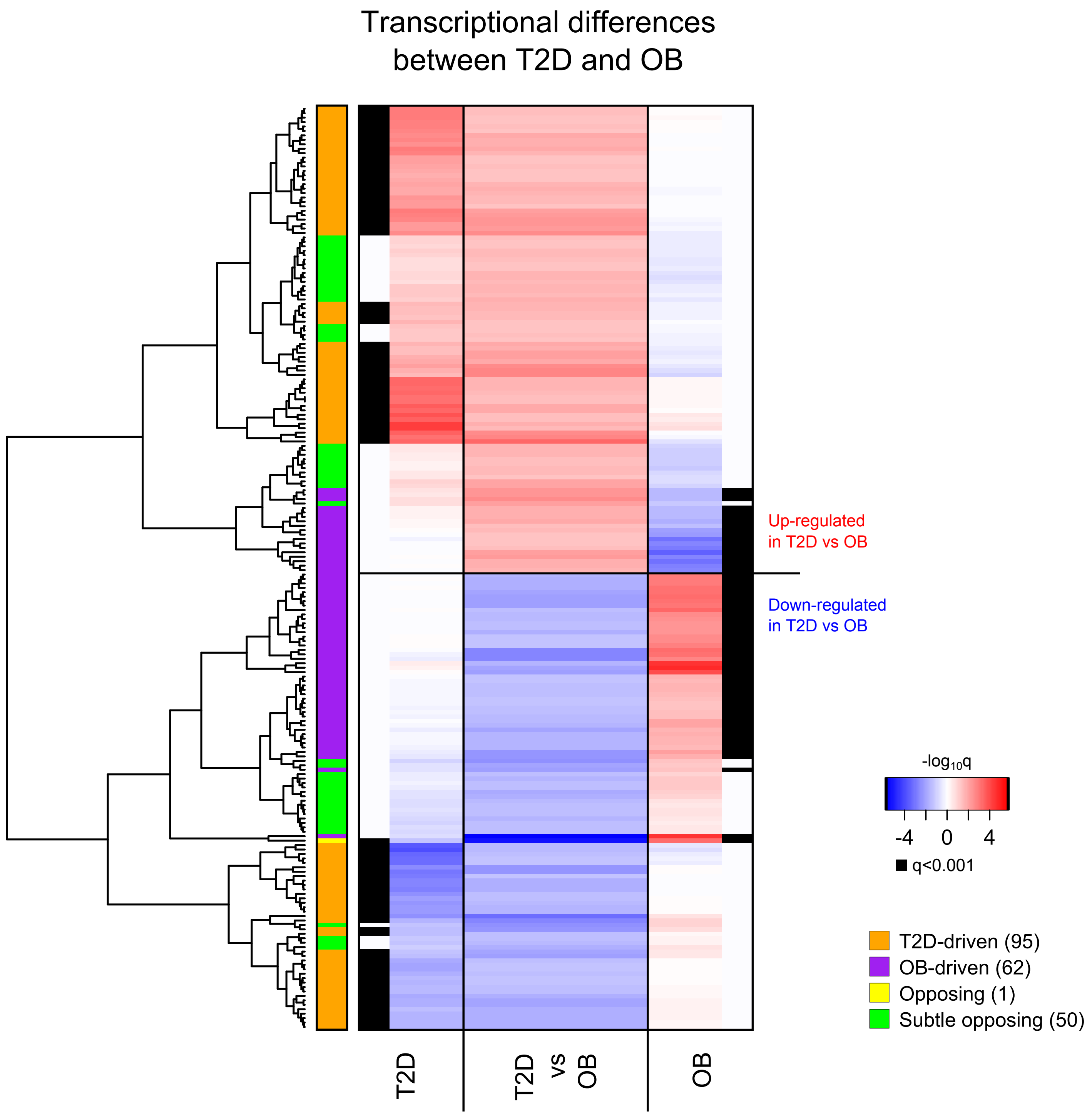

Figure S8. Heatmap showing the q-values (log10-transformed) of genes that were significantly differentially expressed ($q<0.05$) between the T2D and OB groups. The genes were assigned one of 4 classes, depending on their pattern of change. T2D-driven genes (marked orange) are those that were significant in T2D vs controls and non-significant in OB vs controls. OB-driven genes (marked purple) were classified in the same manner as for the T2D-driven genes. Opposing genes (marked yellow) are genes (only one in this case) that were significant in both T2D and OB, vs controls, but with opposite fold changes. Subtle opposing genes (marked green), are the remaining genes, i.e. neither significant in T2D nor OB but showing opposite fold change directions.
